# Measurement of Exam Anxiety Levels Among Medical Students and Their Association With the Influencing Factors

**DOI:** 10.7759/cureus.41417

**Published:** 2023-07-05

**Authors:** Ismail Memon, Ahmad Omair, Omar M Barradah, Nasser M Almegren, Musab M Almuqbil, Omar H Batarfi, Emad Masuadi, Zeeshan Feroz

**Affiliations:** 1 Anatomy, Saba University School of Medicine, The Bottom, BES; 2 Anatomy, College of Science and Health Professions, King Saud Bin Abdulaziz University for Health Sciences, Riyadh, SAU; 3 Anatomy, King Abdullah International Medical Research Center, Riyadh, SAU; 4 Pathology, College of Science and Health Professions, King Saud Bin Abdulaziz University for Health Sciences, Riyadh, SAU; 5 Pathology, King Abdullah International Medical Research Center, Riyadh, SAU; 6 Medicine, King Saud Bin Abdulaziz University for Health Sciences College of Medicine, Riyadh, SAU; 7 Medicine, King Abdullah International Medical Research Center, Riyadh, SAU; 8 Institute of Public Health, United Arab Emirates (UAE) University, Al Ain, ARE; 9 Basic Sciences, College of Science and Health Professions, King Saud Bin Abdulaziz University for Health Sciences, Riyadh, SAU; 10 Pharmacology, King Abdullah International Medical Research Center, Riyadh, SAU

**Keywords:** westside test, medical students, exam, studying, education, anxiety

## Abstract

Introduction

Studying medical science is a demanding task, often leading to exam anxiety among medical students. This study aims to measure anxiety levels among medical students and their relationship with gender, age, grades, study time, year of study, and learning methods.

Methods

It is a cross-sectional study involving third- to sixth-year medical students, who filled in the questionnaire related to the personal data, studying methods, and the Westside Test Anxiety Scale, to estimate the exam anxiety levels before the final examinations of the academic year 2020-2021. Completed questionnaires were reviewed, entered in Microsoft Excel, and analyzed using SPSS.

Results

We found a significant association between gender and high-test anxiety (p < 0.001), with a higher prevalence among females (47.9%) compared to males (22.5%). Although non-significant, its prevalence was higher among the 20 years old (34%) and those with a GPA 4.00-4.49 (37.9%). Anxiety decreased as the students progressed to higher years of studies (37.9% in the third year to only 9.1% in sixth^ ^year, p=0.073), with the lower incidence among those who studied five days or more per week (26.7%) and no significant difference was observed whether students studying in a group or individually. Though insignificant (p=0.754), learning through textbooks was found to be less stressful (29% vs 33%).

Conclusion

Our findings suggest that mediocre and female students are more vulnerable to exam high-test anxiety. Progression to senior years and use of textbooks were associated with lower anxiety levels. A cohort longitudinal study to establish an association between specific factors and anxiety levels is recommended.

## Introduction

The success of a student is mainly judged based on his grades; therefore, a student is pressured to perform well in all types of academic assessments. Usually, a student’s learning environment is test-oriented, in which a test’s outcome dictates many of the psychological states a student pass through. In education, a high level of exam-related anxiety is often experienced by students [1]. A higher prevalence of anxiety and depression has been reported among medical students; particularly, surface learners are more vulnerable leading to either their failure or being less successful [2-4]. Schoolwork and examinations are the most stressful in young adults' lives [5]. Many students become exhausted mentally and physically and cram at night during their exams [6]. The overwhelming emotions, stressful thoughts, and increasing study load, fuel students' anxiety levels. Although some levels of anxiety are necessary for academic success, tremendous feelings of anxiety can hinder students' reasoning abilities and ultimately their academic success [7].

Medical science is a demanding field involving studying, exploring, learning, and continuous assessments. In contrast to other professions, medical students are subject to copious amounts of information to memorize and rigorous testing, which contributes to the students’ psychological state [8]. With different back-to-back examinations, medical students must cover vast materials in a short period of time, compelling them to use various accessible resources [9]. In the United States, medical students in their four-year program must pass their regular compulsory examinations and step-1 of the United States Medical Licensing Examination (USMLE) [10]. Additionally, these back-to-back exams might result in anxiety and thus negatively affect academic performance [11].

The West Side anxiety scale identifies students with anxiety impairment. It is reliable and valid for educational institutes and exam-anxiety evaluations of the students' performance [12]. The GAD-7 scale assesses the severity of generalized anxiety disorder, and the Hamilton anxiety scale assesses a patients’ anxiety severity [12]. All these scales measure anxiety, exam anxiety, and its relation to study methods.

This study aimed to measure anxiety levels influenced by various factors (age, gender, grades, year of the study, time spent on studying, and learning methods) using different scales and to establish a relationship between exam anxiety and the different techniques used by students for preparation. By assessing the anxiety levels among medical students and the associated factors, the institutions can modify curricular and extracurricular activities in order to create either a stress-free or a comparatively less stressful environment that can, in turn, improve the student’s academic performance.

## Materials and methods

Study subjects

All male and female students in the third to sixth academic years at the College of Medicine (COM), were invited to complete the self-administered, anonymous questionnaire by sending an E-form prepared in Google Forms during the academic year 2020-2021.

Questionnaire

A self-developed questionnaire consisted of two parts. The first part of the questionnaire contained demographic questions and specific questions related to the studying methods to assess the utilization of available study resources, time spent on studying, and if the participants were studying in a group. The second part utilized Westside Test Anxiety Scale [12] to estimate the exam anxiety levels. Westside Test Anxiety Scale comprised of ten simple questions to recognize those students who suffered from test anxiety impairment affecting their academic progression. Each question was given a score ranging from 1 (never or not at all) to 5 (extremely or always true), with gradual variations. The total points of the questions were divided by 10. The yielded results were used for interpretations and classification of exam anxiety levels as low (1.0-1.9), normal (2.0-2.5), high normal (2.6-2.9), moderately high (3.0-3.4), high (3.5-3.9), and extremely high anxiety (4.0-5.0). For the data to be more representable, we divided the participants into three main groups based on their test anxiety scores calculated using the Westside scale. Low test anxiety (1.0-2.5), moderate test anxiety (2.6-3.4), and high test anxiety (3.5-5.0).

The respondents in this study could not have differentiated between the two clinical conditions (anxiety and depression) they suffered from during the examination. Therefore, both conditions were studied under the heading of anxiety.

Statistics

The research group members sent an E-survey through electronic links by Google Forms to the students at COM. The data was collected from March 2021 to May 2021 before the final examinations of the academic year 2020-2021. Completed questionnaires were collected and reviewed before entering them into Microsoft Excel and analyzing them using the SPSS software. The study and outcome variables were presented using descriptive statistics (mean, standard deviation, and percentages). Pearson's chi-square test for trend was used to assess and quantify the association between a categorical outcome and different study variables. A test with p-value of <0.05 was considered significant.

Ethical considerations

A consent form was attached to the questionnaire, and participation was entirely optional. Participants had the right to withdraw at any time, and no personal information regarding the participants' identification was required. All the collected data was kept confidential. This research project was approved by the local Institutional Review Board (IRB) at King Abdullah International Medical Research Center, Riyadh, Saudi Arabia (study no. SP20/408/R, ref No IRBC/1915/20, approval ref. No: RYD-20-419812-122633).

## Results

All the male and female students from batch 14 to 17 were invited. We received 246 submissions, out of which 207 were found valid, accounting for only (58.15%) out of the intended 356 students. Table 1 shows the basic characteristics of the study subjects.

**Table 1:** Characteristics of study subjects

		N	%
Gender	Male	138	66.0
	Female	71	34.0
Age	20	50	24.2
	21	78	37.7
	22+	79	38.2
GPA	< 4	18	8.7
	4.00 - 4.49	58	28.0
	4.50 - 5.00	131	63.3
Academic Year	3^rd^	87	41.6
	4^th^	80	38.3
	5^th^	20	9.6
	6^th^	22	10.5
Number of days spent studying per week	Only before exam	31	14.8
	1-2 Days	14	6.7
	3-4 Days	44	21.1
	5 Days or more	120	57.4
Method of study	Alone	199	95.2
	With group	10	4.8

**Figure 1:**
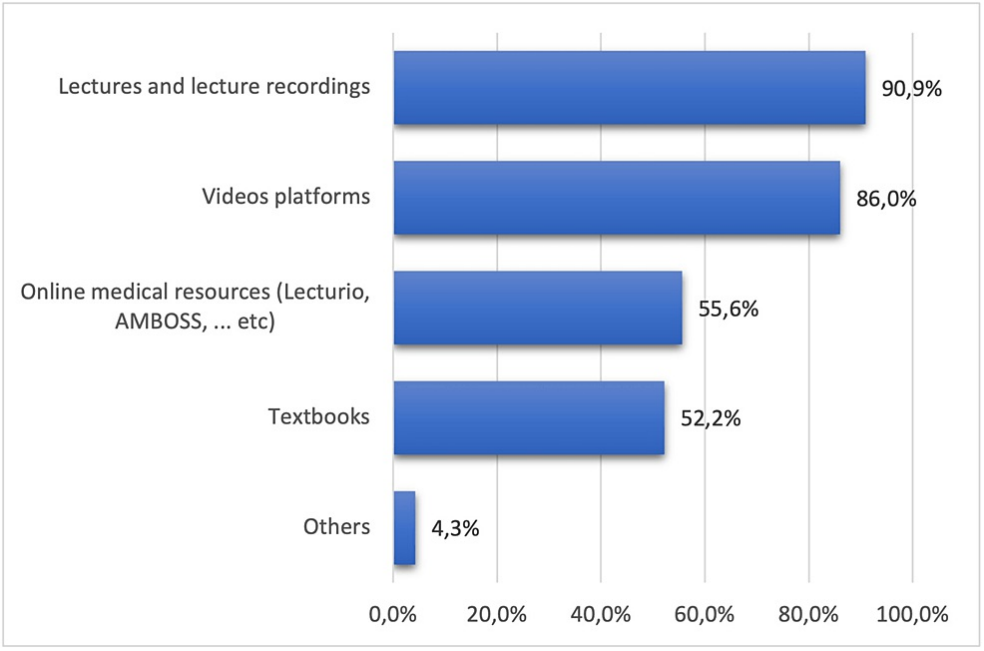
Learning resources used by the students

The proportion of male participants was higher (66%) than females (34%). The lowest number of participants (24.2%) were of 20 years of age, followed by 21 years and 22 years and above (37.7% and 38.2%, respectively). Most of the participants had a GPA of 4.5-5.0 (63.3%), followed by a GPA of 4-4.49 (28%) and those with a GPA of <4 (8.7%). The highest number of respondents were third year students (41.6%), followed by fourth year (38.3%), fifth year (9.6%), and sixth year students (10.5%). Most of the participants (57.4%) spent five days or more studying every week, followed by 39.2% of participants who studied three to four days/week. Only 14.8% of students studied just before the exam. Most of the participants (95.26%) studied alone, whereas only 4.8% studied with a group. Figure 1 shows the studying resources utilized by students.

Most of the students (90.9%) used lectures and lecture recordings, followed by video platforms (86%), online resources (55.6%), and textbooks (52.2%). The levels of test anxiety were not substantially different among the students. The majority of students (36.4%) had low test anxiety, 32.5% had moderate test anxiety, and 31.1% had high test anxiety (Table 2).

**Table 2:** Anxiety levels among the participants Frequencies given as number (N) and percentages (%)

Anxiety classifications		
Low Test Anxiety	76	36.4
Moderate Test Anxiety	68	32.5
High Test Anxiety	65	31.1

Table 3 depicts test anxiety among different groups stratified by different factors.

**Table 3:** Association of anxiety with different factors *A p-value of <0.05 was considered significant.

		Low test anxiety		Moderate test anxiety		High test anxiety		
		N	%	N	%	N	%	P-value
Gender	Male	59	42.8	48	34.8	31	22.5	0.001*
	Female	17	23.9	20	28.2	34	47.9	
Age	20	17	34.0	16	32.0	17	34.0	0.953
	21	28	35.9	25	32.1	25	32.1	
	22+	31	39.2	26	32.9	22	27.8	
GPA	< 4	6	33.3	8	44.4	4	22.2	0.603
	4.00 - 4.49	19	32.8	17	29.3	22	37.9	
	4.50 - 5.0	50	38.2	42	32.1	39	29.8	
Academic year	3^rd^	27	31.0	27	31.0	33	37.9	0.073
	4^th^	31	38.8	22	27.5	27	33.8	
	5^th^	7	35.0	10	50.0	3	15.0	
	6^th^	11	50.0	9	40.9	2	9.1	
No. of days spent studying per week	Only before exam	12	38.7	7	22.6	12	38.7	0.417
	1-2 Days	3	21.4	7	50.0	4	28.6	
	3-4 Days	14	31.8	13	29.5	17	38.6	
	5 Days or more	47	39.2	41	34.2	32	26.7	
Method of study	Alone	72	36.2	65	32.7	62	31.2	0.969
	With group	4	40.0	3	30.0	3	30.0	

The female students (47.9%) were significantly more prone to high test anxiety (p=0.001) compared to the male students (22.5%). Among the different age groups, although statistically insignificant (p=0.953), a higher number of the respondents (34%) with high test anxiety belonged to 20 years. The GPA is a strong indicator of students' ability and academic performance. No statistically significant difference in anxiety levels was observed among students with different GPAs (p=0.603). High test anxiety was more prevalent among the group of students with GPA 4.00-4.49. The groups with GPA of 4.50-5.0 and <4 were comparatively less exposed to high test anxiety. A trend toward significance was observed between the student’s seniority and levels of anxiety (p=0.073). The prevalence of high test anxiety decreased as the students became seniors and went to higher years of studies. The high test anxiety was highest among the third-year students (37.9%), followed by the fourth year (33.8%), fifth year (15%), and sixth-year students (9.1%). No significant association was observed between anxiety levels and number of days of study (p=0.417). High test anxiety was less prevalent among participants who studied five days or more per week (26.7%). The percentage of students with high test anxiety among groups who studied only before the exam, one to two days/week and three to four days/week was 39.3%, 28.6%, and 38.6%, respectively. No significant difference was observed between the number of participants with high test anxiety (p=0.969), who studied alone or in a group (31.2% and 30.0%). For learning methods, most of the participants (90.9%) used lectures and lecture recordings, followed by video platforms (86.0%), online resources (55.6%), and textbooks (Figure 1). Table 4 shows the association between learning resources and anxiety levels.

**Table 4:** Association of anxiety with learning resources *A p-value of <0.05 was considered significant.

		Anxiety						P-value
		Low test		Moderate test		High test		
		N	%	N	%	N	%	
Lectures only	No	73	38.6	57	30.2	59	31.2	0.045*
	Yes	3	15.0	11	55.0	6	30.0	
Lectures and lecture recordings	No	12	60.0	6	30.0	2	10.0	0.037*
	Yes	64	33.9	62	32.8	63	33.3	
Textbooks	No	34	34.0	33	33.0	33	33.0	0.754
	Yes	42	38.9	34	31.5	32	29.6	
Online medical resources (Lecture, AMBOSS etc.)	No	29	31.2	33	35.5	31	33.3	0.348
	Yes	47	40.9	34	29.6	34	29.6	
Videos platforms	No	6	20.7	14	48.3	9	31.0	0.081
	Yes	70	39.1	53	29.6	56	31.3	
Others	No	72	36.0	66	33.0	62	31.0	0.782
	Yes	4	44.4	2	22.2	3	33.3	

Significant associations were observed between anxiety levels for those who preferred lectures and lectures plus recordings (p=0.045 and 0.037, respectively). A large number (55%) of those students who preferred lectures only as their learning resource had moderate test anxiety compared to 30.0% who had high test anxiety. Among the group who studied from lectures as well as lecture recordings, a similar percentage of students (33.9%, 32.8%, and 33.3%, respectively) was observed to have low, moderate and high test anxiety.

Learning from textbooks or from online medical resources caused high test anxiety in only 29.6% of the students. The majority of the students (39.1%) who used video platforms as their learning method had low test anxiety, followed by 29.6% with moderate test anxiety and 31.3% with high test anxiety. Among the students who used other resources as their learning method, the majority (44.4%) had low test anxiety, followed by 22.2% with moderate test anxiety and 33.3% with high test anxiety. No significant association was found between anxiety and use of textbooks, online medical resources, and video platforms. Among the students who used other resources as their learning method, the majority (44.4%) had low test anxiety, followed by 22.2% with moderate test anxiety and 33.3% with high test anxiety.

## Discussion

This study examined exam-related anxiety levels among medical students and their correlation with gender, age, academic grades, academic year, and group study. The majority of the participants were males and those in the third year of medical school; the majority studied five days or more a week, preferred studying alone, and used lectures and lecture recordings. High test anxiety was more prevalent among female students, students with GPA 4.00-4.49, and junior class students, whereas it was less prevalent among those who studied five days or more per week. Significant associations were observed between anxiety levels for those who preferred lectures and lectures plus recordings. Compared to high test anxiety, moderate test anxiety was more prevalent among those who preferred lectures only as their learning resource.

Stress and anxiety share similar emotional and physical symptoms, but they have different origins. Generally, anxiety among medical students has been recorded worldwide with adverse effects on their cognitive function and learning [4,13,14]. Similarly, this study also showed that medical students suffered from low to high test anxiety (Table 2). Various factors responsible for exam-related anxiety have been assessed here.

The number of respondents in this study decreased from the third year to the sixth year. It is common to observe that senior students are less likely to be interested in questioner-based studies, which might be because of their busy schedules and a high load of clinical work. Studying Medicine is highly demanding as most participants in this study studied persistently for four to five hours during weak days and preferred studying alone (Table 1). Usually, group study is helpful in Medicine but because of Covid SOPs, the students in this study chose to study separately which is similar to what has been reported by another study from Saudi Arabia [15].

Many studies worldwide have shown contradictory findings regarding the anxiety level among male and female medical students [3,16-20]. In line with their findings, we also found a significant association of exam-related anxiety with gender (p=0.001). High test anxiety was more prevalent among female medical students (Table 3). Generally, girls are more prone to anxiety because of their thought control strategies and metacognitive beliefs [16]. The other reason for high test anxiety among female students could be that they are meticulous in their endeavors and expect higher academic achievements. Additionally, women in Saudi Arabia are more focused on their home-related chores and children caring than outdoor responsibilities; however, such trends are revolutionary changing now. All these factors cumulatively contribute to a higher level of exam anxiety in female students. Moreover, Cipra et al. and Eum et al. showed that gender had no influence on test anxiety yet significantly impacted exam-related acute anxiety [2,21]. In contrast to our findings, Bashir et al. found that male students experienced higher anxiety levels than females in a Sudanese cohort [5]. The majority of the studies support the fact that female medical students are more vulnerable to developing exam-related anxiety.

It has been previously reported that the test anxiety scores usually decrease with participants’ age as their experience and maturity levels increase [22,23]. In the present study, although there was not a significant association between age and anxiety levels (p=0.953), similar to what has been reported earlier it was observed to be more prevalent among younger age groups (20 years compared to 21 and 22 years).

The published educational literature indicates conflicting opinions about the association of Grade Point Average (GPA) with anxiety levels. Many studies showed an association between low GPA and high test anxiety [4,5], whereas others did not [24-26]. This study showed that the students' GPA was not significantly associated with test anxiety (p=0.603). However, most students with GPA 4.00 to 4.49 showed high test anxiety and the students with higher and lesser GPA were less likely to be exposed to high test anxiety (Table 3). We speculate that mediocre students are more careful of their grades and hence more likely to develop anxiety. The high-GPA students were satisfied with their performance and expected better exam grades. On the other hand, the lesser GPA students were less careful about their academic performance.

The association of study years with anxiety among medical students is not conclusive. Like many previous studies [13,24,27,28], this study also indicated that the students' anxiety levels decreased as they proceeded to higher academic years. But similar to the findings from Dawood et al. [22], this association was not significant (p=0.073). In agreement with findings reported by Zwane et al. we believe that in the initial academic years, the new students are unclear about their learning goals, learning outcomes, study planning, and learning strategies; hence, they suffer from higher levels of anxiety [11]. Additionally, academic, personal, social, financial, and health factors are reported to be countable factors [29]. When the students become seniors in the succeeding years, all these pressures tend to decline [30]. Also, they develop coping strategies with institutional support through student mentorship and buddies’ advisory programs, so their anxiety level also tends to decrease. Conflicting reports also indicate that as medical students proceed to higher academic years, their clinical workload and assignments increase, and consequently their anxiety increases [13,31].

In addition to many other factors contributing to anxiety among medical students, the duration of time spent on studying, i.e., days/week could also relate to exam anxiety. Logically, those students who spend more time studying could have less anxiety, but in this study, we did not find a significant association between the time spent studying and anxiety levels (p=0.417). Nonetheless, of those students who studied for five days or more per week, only 26% had high test anxiety; the remaining participants who gave less time to study were more prevalent to have high test anxiety (Table 3). In line with our findings, previous studies have also not shown any significant association between time spent and test anxiety [32] but agree with the fact that surface learners who give less time studying are more exposed to developing anxiety [2]. These findings and our experiences suggest that the time used for studying could be classified as productive and non-productive or less productive, which depends on many other factors like previous knowledge of the topic, level of concentration, language skills, etc. Less productive time given for studying might cause anxiety.

Our findings of lesser anxiety among textbook users compared to higher anxiety among those using lecture recordings, signify the importance of textbooks and online medical resources as a lesser stressful method of learning. Of course, lectures and lecture recordings are precise, more focused on learning objectives, and easy to memorize. Still, to prepare for an exam, one needs a thorough understanding of the topic, which can only be achieved by studying textbooks and online medical resources. Self-study from textbooks and online resources is an active learning strategy that enhances students’ cognitive learning and decreases exam anxiety. These findings portray that using lectures and lecture recordings as the only method of studying is not an ideal strategy and may become a cause of exam anxiety among students. The didactic lectures were observed to be a cause of moderate anxiety among 55% of students. The lectures could be made more effective if conducted in an interactive manner with a discussion [33,34].

The strengths of this study included its cross-sectional design, participation of both male and female students from three different years of medical school, comprehensive nature of the questionnaire, and use of a reliable method (Westside Test Anxiety Scale) for assessing anxiety levels. On the other hand, the self-reported information provided by students induces a potential for reporting bias which may have occurred because of the respondent's interpretation of the questions or desire to convey their emotions in a certain specific way or simply because of inaccuracies in responses. We recommend that a longitudinal study could be carried out with a cohort of students from all five years of undergraduate medical study, to investigate the levels of exam-related anxiety along with assessing the contributing factors.

## Conclusions

The finding of this study suggests that female students are more prone to higher levels of test anxiety. Usually, mediocre students are more careful of their grades and more likely to develop anxiety. Students' anxiety levels decrease as they proceed to higher academic years. Most of the participants with high exam anxiety used lectures and lecture recordings. Nonetheless, studying regularly, through textbooks and online resources was less stressful. Identifying the major factors causing anxiety could help the universities to modify their curriculum, teaching strategies, and learning resources that will reduce anxiety among medical students and improve their performance. However, this study provided novel information about exam anxiety among Saudi medical students.
